
JOURNAL INFORMATION
==============================
NLM Title Abbreviation: Wirtschaftsdienst
Journal ID: Wirtschaftsdienst
NLM Journal ID: 101086612

Wirtschaftsdienst (Hamburg, Germany : 1949)

ISSN: 0043-6275

ARTICLE INFORMATION
==============================
PMCID: PMC8287282
PMID: 34305186
DOI: 10.1007/s10273-021-2948-8
Article ID: 2948
Article version: 1
Subjects: Leitartikel

Nützt die Schuldenbremse den kommenden Generationen?

Do the fiscal rules benefit future generations?

Breuer Christian c.breuer@zbw.eu

Redaktion Wirtschaftsdienst, ZBW — Leibniz-Informationszentrum Wirtschaft, Neuer Jungfernstieg 21, 20354 Hamburg, Deutschland
Electronic publication date: 2021 Jul 19
Print publication date: 2021
Volume: 101
Issue: 7
First page: 490
Last page: 491
Copyright: © Der/die Autor:in(nen) 2021
License: Open Access: Dieser Artikel wird unter der Creative Commons Namensnennung 4.0 International Lizenz veröffentlicht (https://creativecommons.org/licenses/by/4.0/deed.de).
Open Access wird durch die ZBW — Leibniz-Informationszentrum Wirtschaft gefördert.
License URL: https://creativecommons.org/licenses/by/4.0/

issue-copyright-statement: © The Author(s) 2021

==============================

Die Schuldenbremse sei im Interesse der kommenden Generationen, argumentieren Befürworter. Sie verhindere, dass Mehrheiten überwiegend älterer Wähler eine Politik durchsetzen, die ihnen ermöglicht, sich zulasten künftiger Steuerzahler zu bereichern (Sachverständigenrat, 2007). Wiederwahlorientierte Politiker:innen könnten diesen kurzsichtigen Interessen verfallen und öffentliche Haushalte mit zu hohen Defiziten belasten. Demnach wäre zu erwarten, dass jüngere Wählergruppen tendenziell Parteien unterstützen, die die Schuldenbremse nach der COVID-19-Krise schnell wieder einführen wollen und ältere Wählergruppen Parteien unterstützen, die ihnen bei der Durchsetzung ihrer konsumtiven Interessen behilflich sind und die Schuldenbremse aufweichen bzw. reformieren wollen. Paradoxerweise ist das nicht der Fall. Parteien, die die Schuldenbremse vehement verteidigen, haben eher bei älteren Wählern hohe Zustimmungswerte. Bei der Europawahl 2019 wählten 46,7 % der über 70-Jährigen, aber nur 11,6 % der 18- bis 24-Jährigen die CDU/CSU. Hingegen wählten 34,9 % der 18- bis 24-Jährigen die Grünen, eine Partei, die die Schuldenbremse investitionsorientiert reformieren möchte. Bei den über 70-Jährigen erreichten die Grünen nur 8,6 % der Wähler (Bundeswahlleiter, 2019). Aber auch aktuelle Umfragen bestätigen diese Trends.

Womöglich entspricht die Schuldenbremse in ihrer aktuellen Form nicht den Interessen der kommenden Generation. Hierfür könnten verschiedene Kanäle verantwortlich sein: Makroökonomisch verhindern die Fiskalregeln europaweit eine fiskalpolitische Expansion, was unter anderem für hohe strukturelle Arbeitslosigkeit vor allem in Südeuropa verantwortlich gemacht wird. Dies versperrt bei bestehendem Kündigungsschutz besonders jüngeren Berufseinsteigern als Outsidern auf dem Arbeitsmarkt Einkommensperspektiven. Zudem verschiebt die restriktive Fiskalpolitik in Europa den fiskalisch-monetären Politikmix in Richtung geldpolitischer Expansion durch die EZB, die unter anderem für die aktuelle Vermögenspreisinflation verantwortlich gemacht wird. Hiervon profitieren wiederum überwiegend ältere Immobilien- und Aktienbesitzer, während sich das zu erwartende Arbeitseinkommen der jüngeren relativ dazu reduziert. Ferner führt die Defizitorientierung der Schuldenbremse dazu, dass Anreize gesetzt werden, weniger investive Ausgaben zu tätigen, die besonders in der Zukunft Nutzen stiften, sowie explizite Verschuldung durch implizite, z. B. höhere Rentenversprechen, zu ersetzen, da letztere nicht durch die Schuldenbremse begrenzt wird.

Die wesentliche Kritik der Grünen betrifft die öffentlichen Investitionen. Dass hier Handlungsbedarf besteht, ist weitgehend unstrittig (Dullien et al., 2021; Hüther und Jung, 2021). Da Investitionen bei Kreditfinanzierung das staatliche Nettovermögen unberührt lassen, werden Ausnahmen von der Defizitbegrenzung für Investitionen vorgeschlagen (Breuer, 2021). Gerade wenn der Regierungsmehrheit unterstellt wird, dass sie Belastungen in die Zukunft transferieren möchte, dürfte sie versucht sein, Investitionen zu vermeiden (Hellwig, 2021). Dies betrifft auch die heute relevanten Investitionen in Digitalisierung und die Energie- und Verkehrswende. Politökonomisch ist denkbar, dass die Defizitorientierung nicht dem Interesse der kommenden Generation entspricht, sondern vielmehr denjenigen, die heute sinnvolle und notwendige öffentliche Investitionen verhindern möchten, z. B. Lobbys der Energie- und Automobilindustrie, die wenig Interesse an einer forcierten Energie- und Verkehrswende haben könnten.

Häufig wird angemerkt, der Investitionsbegriff sei unklar (u.a. Bachmann, 2021). Diese Kritik gilt jedoch auch für den Defizitbegriff. Natürlich würde, ebenso wie das Defizit nur einen Teil der Bruttostaatsverschuldung begrenzen kann (explizite, nicht implizite), eine Ausnahme für Investitionen, etwa für Bau- und Anlageinvestitionen, nur einen Teil des öffentlichen Kapitalstocks betreffen, während andere investive Ausgaben unberücksichtigt blieben. Letztere würden zwar weiterhin mit rein konsumtiven Ausgaben gleichgestellt, aber die gezielte Förderung einzelner klar investiver Ausgaben ist besser als die Vernachlässigung aller Investitionen.

Die Abschaffung oder Reform der deutschen Schuldenbremse erfordert eine Änderung des Grundgesetzes, also eine Zweidrittelmehrheit in Bundestag und Bundesrat. Dabei stellt sich die eher philosophische Frage, weshalb angenommen wird, dass einer einfachen Parlamentsmehrheit nicht zugetraut wird, eine nachhaltige Haushaltspolitik zu betreiben, eine Zweidrittelmehrheit der gleichen Parlamente aber das Interesse der kommenden Generationen vertreten könne. Die für eine Verschärfung der Schuldenbremse erforderlichen zusätzlichen Stimmen aus der Opposition könnten sich z.B. ergeben, wenn eine kurzfristorientierte Opposition der Regierung misstraut und den Spielraum der Regierung einengen möchte, unabhängig davon ob die Regelung im Detail im Interesse der kommenden Generationen ist. So wurde die Unterstützung der SPD zur Schuldenbremse auch damit begründet, dass darin eine „Steuersenkungsbremse" gesehen wurde, die eine sich damals in Umfragen abzeichnende Koalition aus CDU/CSU und FDP dabei beschränkte, angekündigte Steuersenkungen umzusetzen (Bluth, 2016, 130).

Sollte eine Verfassungsänderung bis zur Wiedereinführung (wohl erst 2023) nicht zustande kommen, was wahrscheinlich ist, dürften reformwillige Regierungen eine Verfassungsänderung mit einfachgesetzlichen Mitteln zu substituieren suchen. So würde die Schuldenbremse länger als erwartet ausgesetzt bleiben, was ihre Wiedereinführung erschwert (Büttner, 2021). Investive Extrahaushalte könnten die Problematik der bisher nicht-investiven Ausgestaltung kompensieren (Hüther und Südekum, 2021; Südekum, 2021). Veränderte Berechnungen der Konjunkturkomponente könnten die Kreditspielräume erhöhen (Südekum, 2021; Krahé und Sigl-Glöckner, 2021). Die Reform des Fiskalpakts auf EU-Ebene ist ebenso in der Diskussion und erfordert keine Änderung des Grundgesetzes (Truger, 2020; Hauptmeier und Leiner-Killinger, 2020). Eine Umfrage unter internationalen Wissenschaftlern vom Juni 2021 zeigte, dass 98 % der Befragten eine Reform der Fiskalregeln als notwendig erachten (CFM Surveys, 2021). Die Parlamente sollten diese Stimmen aus der Wissenschaft sowie die aufgedeckten Präferenzen junger Menschen ernst nehmen.

Christian Breuer ist Chefredakteur der Zeitschriften Wirtschaftsdienst und Intereconomics in der ZBW — Leibniz-Informationszentrum Wirtschaft in Hamburg.


Literatur

Bachmann R Bidens Fiskalpolitik — ein Vorbild für Deutschland? Wirtschaftsdienst 2021 101 6 414 417 10.1007/s10273-021-2934-1 34176984 PMC8218272
Bluth, C. (2016), The Political Economy Of National Fiscal Rule Reform in Eu Countries, Cambridge.
Breuer C Staatsverschuldung nach Corona: Rückkehr zur Goldenen Regel Wirtschaftsdienst 2021 101 1 2 3 10.1007/s10273-021-2809-5 33487763 PMC7813608
Büttner T Finanzplanung: Die Krise hat Konjunktur Wirtschaftsdienst 2021 101 7 6
Bundeswahlleiter (2019), Europawahl 2019, 4: Wahlbeteiligung und Stimmabgabe nach Geschlecht und Altersgruppen (http://bundeswahlleiter.de).
CFM Surveys (2021), Fiscal Rules in the European Monetary Union ∣ The CFM-CEPR Surveys, http://cfmsurvey.org.
Dullien S Rietzler K Tober S Öffentliche Investitionen im Konjunkturprogramm als Einstieg in die sozial-ökologische Transformation Wirtschaftsdienst 2021 101 3 172 175 10.1007/s10273-021-2869-6 33746302 PMC7964514
Hauptmeier S Leiner-Killinger N European Central Bank Reflections on the Stability and Growth Pact’s Preventive Arm in Light of the COVID-19 Crisis Intereconomics 2020 55 5 296 300 10.1007/s10272-020-0919-8
Hellwig M Deutschland braucht ein Investitionskompetenzprogramm Wirtschaftsdienst 2021 101 3 176 179 10.1007/s10273-021-2870-0
Hüther M Südekum J Schuldenbremse nach der Coronakrise Wirtschaftsdienst 2020 100 10 746 752 10.1007/s10273-020-2757-5
Hüther M Jung M Unzureichende Investitionsoffensive Wirtschaftsdienst 2021 101 3 158 161 10.1007/s10273-021-2866-9
Krahé M Sigl-Glöckner P Die Definition einer zukunftsfähigen Finanzpolitik Wirtschaftsdienst 2021 101 7 497 500
Sachverständigenrat (2007), Staatsverschuldung wirksam begrenzen — Expertise im Auftrag des Bundesministers für Wirtschaft und Technologie.
Südekum J In der Fiskalpolitik sind Grundsatzentscheidungen notwendig Wirtschaftsdienst 2021 101 6 425 428 10.1007/s10273-021-2936-z
Truger A Reforming EU Fiscal Rules: More Leeway, Investment Orientation and Democratic Coordination intereconomics 2020 55 5 277 281 10.1007/s10272-020-0915-z
